# Expert opinion on patient journey, diagnosis and clinical monitoring in acid sphingomyelinase deficiency in Turkey: a pediatric metabolic disease specialist's perspective

**DOI:** 10.3389/fped.2023.1113422

**Published:** 2023-06-26

**Authors:** Nur Arslan, Mahmut Coker, Gulden Fatma Gokcay, Ertugrul Kiykim, Halise Neslihan Onenli Mungan, Fatih Ezgu

**Affiliations:** ^1^ Division of Pediatric Metabolism, Department of Pediatrics, Dokuz Eylul University Faculty of Medicine, Izmir, Türkiye; ^2^ Division of Pediatric Metabolism, Department of Pediatrics, Ege University Faculty of Medicine, Izmir, Türkiye; ^3^ Division of Pediatric Metabolism, Department of Pediatrics, Istanbul University Istanbul Faculty of Medicine, Istanbul, Türkiye; ^4^ Division of Pediatric Metabolism, Department of Pediatrics, Istanbul University Cerrahpasa Faculty of Medicine, Istanbul, Türkiye; ^5^ Division of Pediatric Metabolism, Department of Pediatrics, Cukurova University Faculty of Medicine, Adana, Türkiye; ^6^ Division of Pediatric Metabolism and Pediatric Genetics, Department of Pediatrics, Gazi University Faculty of Medicine, Ankara, Türkiye

**Keywords:** Niemann-Pick disease, acid sphingomyelinase deficiency, diagnostic delay, diagnostic algorithm, index of suspicion, prognosis

## Abstract

This review by a panel of pediatric metabolic disease specialists aimed to provide a practical and implementable guidance document to assist clinicians in best clinical practice in terms of recognition, diagnosis and management of patients with acid sphingomyelinase deficiency (ASMD). The participating experts consider the clinical suspicion of ASMD by the physician to be of utmost importance in the prevention of diagnostic delay and strongly suggest the use of a diagnostic algorithm including/starting with dried blood spots assay in the timely diagnosis of ASMD in patients presenting with hepatosplenomegaly and a need for increased awareness among physicians in this regard to consider ASMD in the differential diagnosis. In anticipation of the introduction of enzyme replacement therapy, raising awareness of the disease among physicians to prevent diagnostic delay and further investigation addressing natural history of ASMD across the disease spectrum, potential presenting characteristics with a high index of suspicion, as well as biomarkers and genotype-phenotype correlations suggestive of poor prognosis seem important in terms of implementation of best practice patterns.

## 1. Introduction

Niemann-Pick disease (NPD) is an eponym that refers to two distinct metabolic abnormalities including the deficiency of acid sphingomyelinase (ASM) enzyme [NPD types A, A/B and B; collectively called as ASM deficiency (ASMD)] and the defective function in cholesterol transport (NPD type C) (1–3). In ASMD, patients have mutations in the sphingomyelin phosphodiesterase 1 gene (SMPD1 gene) encoding ASM, while NPD type C is a distinct form that differs from ASMD in terms of genetic, pathologic and prognostic considerations (4, 5).

The deficient hydrolysis of sphingomyelin to ceramide and phosphocholine due to deficiency lysosomal enzyme ASM leads to visceral disease and/or neurodegeneration with progressive accumulation of sphingomyelin in multiple organs (liver, spleen, lung, bone marrow, and lymph nodes) (1, 6–8). The birth prevalence of ASMD is estimated at 0.4–0.6/100.000, and while NPD type A is considered to be more common in individuals of Ashkenazi Jewish ancestry, both type A and type B forms are pan ethnic (6, 9).

In 2018, a new terminology for ASMD phenotypes has been proposed including infantile neurovisceral ASMD (NPD type A), chronic neurovisceral ASMD (intermediate form, NPD type A/B) and chronic visceral ASMD (NPD type B) (6). This terminology is considered to reflect the broad spectrum of clinical presentations more accurately (visceral and/or neurological involvement) and disease severity (ranging from a rapidly progressive infantile neurovisceral disease to more slowly progressive chronic neurovisceral and chronic visceral forms) of ASMD, which also contributes to well-known diagnostic challenges (6, 10).

Patients with chronic ASMD suffer from significant morbidity due to multisystemic involvement with consideration of liver dysfunction, hepatosplenomegaly, infiltrative lung disease, and thrombocytopenia as the main causes of death (5, 11–14).

Indeed, the diagnosis of ASMD is often delayed not only because of a diagnostic dilemma created by the heterogeneous clinical presentation but also due to its rarity and insufficient awareness about the disease among physicians (6). Currently, olipudase alfa, a recombinant human ASM, is the only disease-specific treatment for ASMD, which is recently approved by the FDA and EMA as well as in other countries such as Japan and Brazil for the treatment of the chronic, systemic, non-neurologic manifestations of the disease (7, 12, 15). Hence, given the availability of enzyme replacement therapy (ERT), improved awareness of the disease among healthcare professionals and better understanding of the natural history of ASMD across the heterogeneous disease spectrum have become increasingly important for appropriate clinical decision-making (5).

The proposed expert opinion was therefore prepared by a panel of pediatric metabolic disease specialists from Turkey to review the current knowledge on clinical manifestations, diagnosis and monitoring of ASMD and to provide a practical guidance document to facilitate disease awareness in anticipation of the introduction of ERT and to assist clinicians for best clinical practice in terms of early recognition of the disease and timely provision of referrals to improve diagnosis, monitoring and care of patients with ASMD.

## 2. Methods

The present expert panel of pediatric metabolic disease specialists with long-term experience in the management of ASMD convened at a 1-day meeting, supported by the sponsor (Sanofi Turkey), to develop an expert opinion on patient journey, diagnosis and clinical monitoring of ASMD. The panel critically analyzed recommendations from international guidelines and the published studies focusing on ASMD and agreed on a series of statements supported by scientific evidence and expert clinical opinion to assist clinicians in real-life practice. The proposed expert opinion planned to provide a practical guidance document addressing (a) clinical spectrum and natural course of disease, (b) clinical manifestations (pediatric and adult population, disease subtypes), (c) diagnostic odyssey of the ASMD (diagnostic delay, patient journey, diagnostic algorithm), (d) diagnostic laboratory tests, and (e) disease monitoring and management (monitoring assessments, biomarker assays, treatments/interventions/life style modifications).

## 3. Clinical spectrum and natural course of disease

ASMD is a multi-system disease with a wide spectrum of clinical manifestations despite the uniform underlying mechanism of deficient enzyme (ASM) activity (5, 7, 15). Accordingly, there is a continuum of disease severity as driven by concomitant neurological involvement, the extent of systemic disease, the rate of disease progression and the heterogeneity of SMPD1 mutations (5, 7, 15).

Infantile neurovisceral ASMD type A is the severest form characterized by symptom onset in early infancy, rapidly progressive neurodegeneration, progressive psychomotor retardation (generally normal until 6 months of age, plateaus from 6 to 15 months as followed by a rapidly progressive deterioration), progressive systemic manifestations (i.e., pulmonary involvement, hepatosplenomegaly, hypotonia, and failure to thrive), and death by 3 years of age often due to respiratory failure following infection (1, 5–8, 15–17) (Table 1).

**Table 1:** Types of ASMD in relation to symptom onset, phenotype, and prognosis (1, 5, 7).

**Infantile neurovisceral ASMD (ASMD type A)**		
**Age at symptom onset**	**Phenotype**	**Prognosis**
Early infancy	Infantile onset of severe neurodegeneration with progressive psychomotor deterioration	Most severe form Rapidly progressive multisystem disease Death by 3 years of age due to respiratory failure following infection
**Chronic neurovisceral ASMD (ASMD type A/B)**		
**Age at symptom onset**	**Phenotype**	**Prognosis**
From childhood to adulthood	Type B phenotype plus progressive neurologic findings including ataxia, variable degrees of developmental delay and peripheral neuropathy	Intermediate form Slower progression of neurological symptoms and prolonged survival compared to ASMD type A Similar or more severe progressive multisystem disease manifestations than in ASMD type B Premature death from liver and respiratory disease; age at death ranges from childhood to adulthood
**Chronic visceral ASMD (ASMD type B)**		
**Age at symptom onset**	**Phenotype**	**Prognosis**
From childhood to adulthood	Chronic progressive multisystem disease with no or little neurologic involvement	Slowly progressive form A normal life span or die prematurely from disease complications that include respiratory failure, liver failure, and/or hemorrhage Progressive splenomegaly may result from deposition of sphingomyelin and progressive portal hypertension

Chronic forms, including chronic neurovisceral (type A/B) and chronic visceral (type B) ASMD are more slowly progressing forms that can manifest from infancy to adulthood (6, 7, 15, 18–20).

Chronic neurovisceral ASMD (ASMD type A/B) is the intermediate form with onset of symptoms in childhood through adulthood, more slowly progressing neurodegeneration (ataxia, gross motor delays, neurocognitive delay, hypotonia, and peripheral neuropathy and learning disabilities) and prolonged survival compared to ASMD type A, whereas with similar or more severe progressive multisystem disease manifestations than ASMD type B along with shortened life expectancy due to respiratory and/or liver disease (5–7, 11, 14, 15, 18–20) (Table 1).

Chronic visceral ASMD (ASMD type B) is characterized by onset of symptoms from early childhood to adulthood and slowly progressive multisystem disease manifestations (i.e., hepatosplenomegaly, pro-atherogenic lipid profile, delayed growth and puberty, fatigue, bone and joint pain, osteopenia, interstitial lung disease and pulmonary infections) (6, 11, 13, 14, 17, 18). In these patients, a normal life span or premature death from disease complications (i.e., liver failure, respiratory failure, and/or hemorrhage) is possible (11, 14) (Table 1).

## 4. Clinical manifestations of ASMD: presenting symptoms/signs

Acid sphingomyelinase deficiency is associated with impaired homeostatic cycle of cell-membrane phospholipid regulation due to failure of sphingomyelin to be hydrolyzed to phosphorylcholine and ceramide (1, 15). This leads to accumulation of sphingomyelin in almost every cell type, particularly in lipid-laden macrophages, affecting the liver, spleen, lung, bone marrow, skin, and lymph nodes as well as the central and peripheral nervous system in more severe cases (1, 15).

### 4.1. Hepatomegaly and splenomegaly

Hepatosplenomegaly is evident at the time of diagnosis in majority of ASMD patients, with clinically significant liver disease in as high as 40% of patients with chronic forms (5, 13, 14, 17, 21).

Hepatomegaly in ASMD is related to massive infiltration of hepatic sinusoidal spaces by sphingomyelin-laden hepatic macrophages (Kupffer cells) and lysosomal sphingomyelin accumulation in hepatocytes (7).

Liver disease is commonly noted in chronic ASMD patients, as associated with liver failure, cirrhosis or need for liver transplant in some individuals (11). Liver failure, in addition to respiratory disease, is the leading cause of mortality in chronic forms of ASMD (14).

Splenomegaly is also one of the most common disease manifestations (13, 17, 18). Although infiltration of lipid-laden macrophages is the initial cause of splenomegaly (2, 22), more rapid progression of splenomegaly with spleen volumes >20 multiples of normal is considered to occur in relation to worsening portal hypertension resulting from the progression of fibrotic liver disease, and to increase the risk of bleeding, splenic rupture, and mortality (7, 14, 15). Spleen volume is considered to be a surrogate marker of disease severity as correlated with other disease parameters (i.e., hepatomegaly, growth, lipid profile and hematologic parameters) along with-it significant impact on patient quality of life (QoL) by compromising respiratory function and limiting daily activities via massive abdominal distension (5, 11, 13, 15, 17, 23, 24).

### 4.2. Pulmonary disease

Pulmonary involvement refers to one of the key characteristics of the multisystem manifestation of sphingomyelin storage, with radiographic evidence of infiltrative lung disease (ILD) in most patients with ASMD (25–27). The progressive restriction of lung volumes and impaired gas exchange occurs due to accumulation of lipid-laden macrophages in the alveolar septa, bronchial walls, and pleura (7, 28). Hence, in patients with functional pulmonary disease, in accordance with the restrictive lung disease and impaired gas exchange secondary to ILD, the decrease in forced vital capacity and in the percent diffusion capacity of carbon monoxide (DLCO) are commonly noted (5, 15).

Notably, lung-only involvement—without organomegaly—has also been reported in adult ASMD patients (29) as well as the absence of overt respiratory symptoms despite identification of typical reticulonodular patterns of infiltration on chest radiography (26). In addition, dissociation between the extent of ILD on imaging and the degree of lung compromise on pulmonary function test has frequently been noted (26). Accordingly, identification of markedly abnormal imaging findings is possible in case of only mild to moderately impaired gas exchange as well as the detection of mildly abnormal imaging findings in case of marked gas exchange impairment (DLCO <60% of predicted value) (26). Therefore, imaging studies are considered not to be sufficient in the assessment of pulmonary disease in ASMD type B and suggested to be interpreted as accompanied with the functional testing and the clinical status (5). The infiltrative pulmonary process is typically progressive and strongly related to disease burden and severity of sequela (11, 14, 15, 17, 27, 30, 31), while respiratory infections and respiratory failure account for the major causes of mortality in patients (pediatric and adult) with chronic forms of ASMD (5, 11, 14, 15).

### 4.3. Atherogenic lipid profile

Most patients with ASMD have an atherogenic lipid profile including low HDL cholesterol (HDL-C) and elevated levels of LDL-C, VLDL-C and triglyceride (3, 13, 18, 32). A similar lipid profile is also noted in lysosomal acid lipase deficiency (LALD), and low levels of HDL-C also occur in patients with Gaucher disease, while more severe decreases occur in ASMD patients (6). In addition, accelerated atherosclerosis with hypertrophy of medial and intimal smooth muscle cells in distal branches of coronary arteries (32) as well as established coronary artery or heart valve disease (in ∼10% of patients) (11) have been reported in chronic visceral ASMD patients.

### 4.4. Skeletal involvement

Pediatric and adult patients with chronic visceral ASMD commonly suffer from joint and limb pain, while lower bone mineral content (BMC) and bone mineral density (BMD) in spine, hip, and femur in the pediatric setting, and osteopenia or osteoporosis in adult ASMD patients have also been reported (7, 13, 24).

### 4.5. Growth delay

Growth delay is considered likely in children and adolescents with chronic visceral ASMD, along with delayed skeletal age, short stature, low weight, and delayed puberty (7, 13, 23).

Growth delay is most pronounced in adolescents accompanied with delayed bone age and delayed puberty, whereas the height is in low normal range in most adults (≥18 years of age) (5, 13). Hence, while short stature is a cause of concern in the adolescence age, final adult heights approach normal values, suggesting the likelihood of a period of catch-up growth in late adolescence and/or early adulthood in patients chronic visceral ASMD (5, 13).

### 4.6. Neurological involvement

The neurological symptoms are considered to be predominant, absent and variable (ranging from mild hypotonia and hyporeflexia to severe involvement including loss of motor function and cognitive decline) in patients with infantile neurovisceral, chronic visceral and chronic neurovisceral ASMD, respectively (7).

In a study among patients with infantile neurovisceral ASMD, hepatosplenomegaly was reported to be identifiable at 2–4 months of age, while developmental arrest followed by rapidly progressing neurodegeneration started from 7 months of age along with failure to thrive by 10 months of age, respiratory symptoms by 9 months of age, irritability and macular cherry-red spots detectable by 12 months of age and death by 27 months of age (8). Hydrocephalus and magnetic resonance imaging (MRI) findings of delayed myelination, widening of the anterior horn of the left ventricle and an arachnoid cyst was also reported (8).

The childhood vs. infantile period in chronic neurovisceral ASMD has been associated with later onset and slower progression neurological symptoms (7). In chronic neurovisceral ASMD, normal developmental milestones persist at least during the first 2 years of life along with a range of symptoms from mild hypotonia and hyporeflexia to severe involvement (i.e., loss of motor function and cognitive decline) (5, 7, 19, 33). The presence of macular cherry-red spots and Q292K mutation is linked to neurological involvement in patients with chronic ASMD (19, 20, 33).

The present expert panel considers the most common presenting symptoms and signs in both pediatric and adult patients with ASMD to include hepatosplenomegaly, elevated transaminases, jaundice, thrombocytopenia, progressive pulmonary dysfunction, dyspnea, and pulmonary infections. Cherry-red maculae, developmental delay, feeding problems, failure to thrive and hypotonia in the pediatric age, while fatigue, limb/joint pain, neuropsychiatric symptoms, and low BMD in the adult age are also amongst the manifestations (Table 2).

**Table 2:** Presenting symptoms and signs according to patient age.

Most common presenting symptoms and signs in ASMD patients	
Pediatric patients	Adult patients
Hepatosplenomegaly Abnormal LFTs Thrombocytopenia Progressive pulmonary dysfunction, dyspnea Pulmonary infections Cherry-red spot/maculae	
Developmental delay Feeding problems Failure to thrive Hypotonia	Fatigue Limb/joint pain Neuropsychiatric symptoms Low bone mineral density

**Figure 1:**
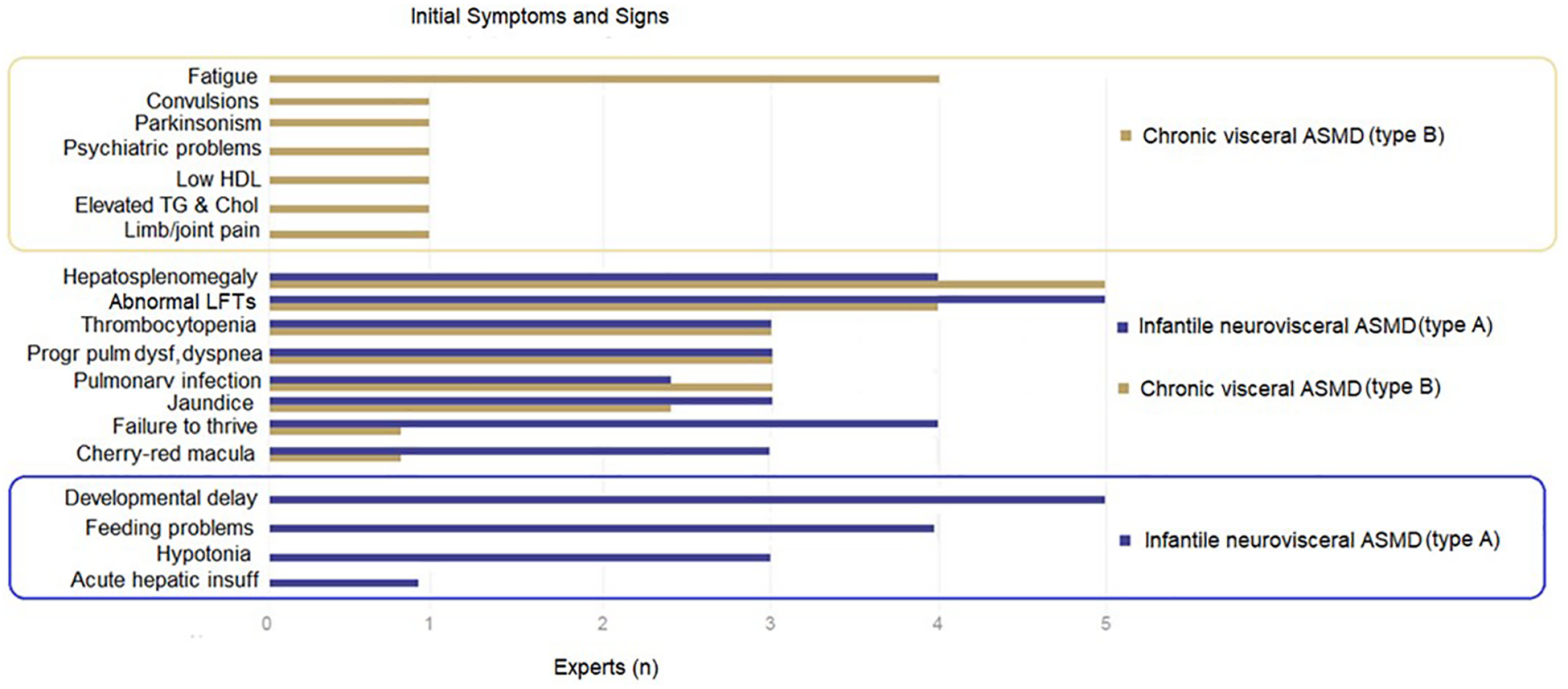
Presenting symptoms and signs according to ASMD types.

For ASMD subtypes, the participating experts consider the developmental delay to be specific to ASMD type A and fatigue to be more common in ASMD type B, while hepatosplenomegaly, abnormal liver function tests, pulmonary dysfunction and cherry-red macula are considered to be prevalent in both types (Figure 1).

## 5. Diagnostic odyssea-patient journey

### 5.1. Diagnostic delay

Frequent misdiagnosis or delayed diagnosis remains a challenge in clinical practice due to lack of awareness of LDSs, including ASMD, among physicians (34–36).

The participating experts consider the suspicion of the disease by clinicians as the key factor in establishing the diagnosis of ASMD, given the availability of appropriate blood-based enzymatic diagnostic testing and genetic sequencing. In addition, while general practitioners and family medicine, general pediatricians, internists, and neurology specialists are also involved in the patient journey as first-admission or referring physicians before the diagnosis of ASMD, the pediatric/adult gastroenterology and hematology specialists seem to have the greatest potential to come across a patient with ASMD deficiency presenting with hepatosplenomegaly and thus in preventing the diagnostic delay by referring these patients directly to pediatric metabolic disease specialists for confirmation of diagnosis, follow-up and treatment. Of note, pediatric metabolic disease specialists in Turkey are assigned to take care of both pediatric and adult patients (Table 3).

**Table 3:** Top-five specialties at first admission or referral until diagnosis.

	Top-five specialties			
	First admission		Referral	
	Pediatric patients	Adult patients	Pediatric patients	Adult patients
1	Pediatrics Pediatric Gastroenterology	Internal Medicine Family Medicine, GP Gastroenterology Neurology	Pediatrics Family Medicine, GP Pediatric Gastroenterology Pediatric Metabolic Disease	Family Medicine, GP Internal Medicine
2	Pediatric Hematology Pediatric Gastroenterology Pediatric Neurology Pediatric Metabolic Disease	Hematology Internal Medicine	Pediatric Hematology Pediatrics Pediatric Neurology	Internal Medicine Hematology Pediatric Metabolic Disease
3	Pediatrics Pediatric Gastroenterology Pediatric Neurology	Gastroenterology Endocrinology Pediatric Metabolic Disease	Pediatric Gastroenterology Pediatrics	Internal Medicine Hematology Gastroenterology
4	Family Medicine, GP Pediatric Gastroenterology Pediatric Hematology	Family Medicine Cardiology Neurology Endocrinology	Pediatric Hematology Family Medicine, GP Pediatric Endocrinology	Endocrinology Cardiology Family Medicine, GP
5	Pediatric Neurology Pediatric Metabolism	Family Medicine Internal Medicine Pediatric Metabolism Pulmonology	Pediatric Neurology Pediatric Pulmonology	Internal Medicine Family Medicine, GP Pediatric Metabolism

Although DBS assay is readily available for any physician who suspect presence of a LSD in his/her patient, given the likelihood of false-negative/positive test findings on DBS assay the diagnosis should be confirmed or ruled out by enzyme assay in leukocytes or by molecular analysis.

Increased awareness among gastroenterologists and hematology specialists, as the most consulted specialists, as well as non-disease-expert physicians seems to be the key factor in consideration of ASMD.

### 5.2. Raising the index of suspicion—a proposed diagnostic algorithm

Misdiagnosis of ASDM at the time of initial presentation is likely due to rarity of disease and the heterogeneity of clinical manifestations (5, 7). Diagnostic guidelines have been published for infantile (neurovisceral), childhood (chronic neurovisceral and chronic visceral) and later-onset (chronic visceral) ASMD, in accordance with the most prevalent symptoms at initial presentation, the associated symptoms predictive of ASMD, the differential diagnoses, and the diagnostic testing paradigms (6) (Tables 2, 3).

**Figure 2:**
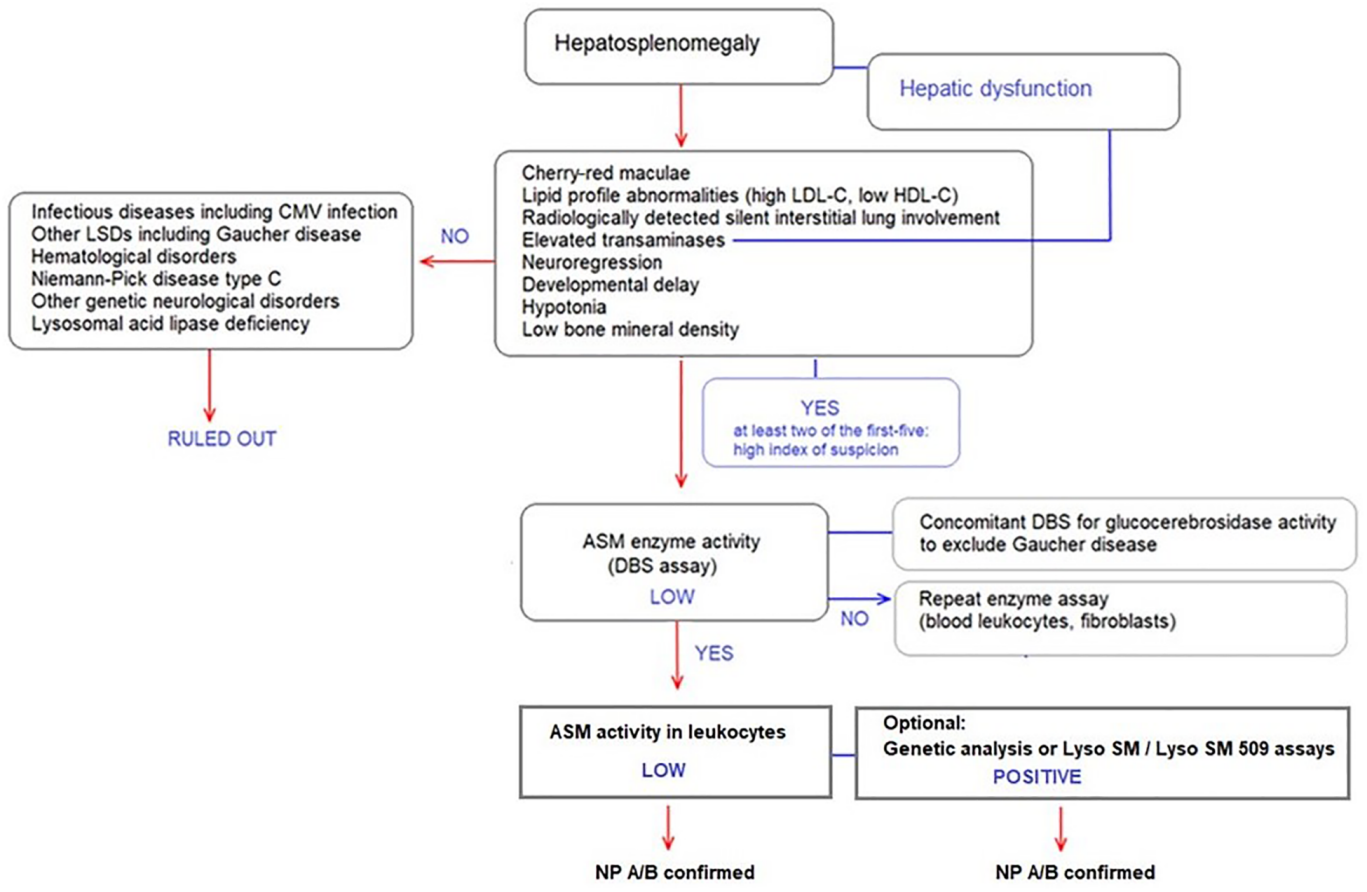
Diagnostic algorithm for ASMD type A, A/B and B in patients presenting with hepatosplenomegaly.

The proposed diagnostic algorithm is provided in Figure 2.

Specifically, ASMD should be considered in the diagnosis of patients presenting with hepatosplenomegaly. Presence of at least two of the following in a patient presenting with hepatosplenomegaly refers to high index of suspicion (Figure 2):

• Cherry-red maculae
• Lipid profile abnormalities (i.e., high LDL-C, low HDL-C),
• Radiologically detected silent interstitial lung involvement
• Elevated transaminases
• Neuroregression

In the presence of signs and symptoms with high index of suspicion for ASMD, DBS assay to test ASM enzyme activity should be performed, as followed by enzyme assay in leukocytes with Lyso-SM and Lyso-SM 509 tests where available. Genetic analysis of the *SMPD1* gene may also be used to confirm the diagnosis. The experts consider simultaneous determination of ASM and glucocerebrosidase activities in DBS followed by enzyme assay in leukocytes for any patient referred with a preliminary diagnosis of “Gaucher Disease” as there is a significant clinical overlap (37) (Figure 2).

Infectious diseases including CMV infection, other LSDs including Gaucher disease, hematological disorders, NPD type C, other genetic neurological disorders and LALD should be considered in the differential diagnosis of a patient presenting with hepatosplenomegaly (Figure 2).

The participating experts consider preparation of simple “red flag” cards based on the most frequent symptoms of the disease that would remind the physicians to consider ASMD in differential diagnosis of a patient with unexplained hepatosplenomegaly as well as implementation of awareness raising projects via ASMD-based educational and informative meetings may help to improve disease awareness among physicians.

## 6. Diagnostic laboratory tests

### 6.1. Gold standard tests

Enzyme assay in leukocytes to quantify ASM activity is the gold standard for the diagnosis of ASMD. Enzyme assay in DBS has been used as an easy and rapid method to “screen” patients with suspected ASMD, the possible false negative/positive results with this method necessitated confirmation with enzyme assay in leukocytes.

### 6.2. Molecular genetic testing and phenotype-genotype correlations

Molecular testing, generally DNA sequence analysis of the SMPD1 gene is generally referred after coming across a low ASM activity either in DBS or leukocytes (1, 38–42).

While ASMD is a pan-ethnic genetic disease, phenotypic differences are likely given that many mutations are private and others are found preferentially in specific ethnic groups (5). Hence, demonstration of missing or significantly diminished enzyme activity is of critical importance, given the existence of many unique mutations or genetic variants of unknown significance (VUS) and most genetic variants are not pathogenic (6).

Infantile neurovisceral ASMD (type A) is associated with p.R498l, p.L304P, and p.P333Sfs*52 variants [homoallelic for p.R498l, p.L304P and p.P333Sfs*52 (Ashkenazi founder mutations)]. Chronic neurovisceral ASMD (type A/B) is associated with p.Q294K and p.W393G variants. Chronic visceral ASMD (type B) is associated with p. ΔR610, p.P323A, p.P330R, and p.W393G variants [homo- or heteroallelic p. ΔR610, p.P325A, and p.P332R (neuroprotective); p.W393G]. For unknown genotype/phenotype correlations, clinical assessment is needed to determine phenotype (6).

### 6.3. Newborn screening

Newborn screening for ASMD is feasible by testing the enzymatic activity in DBS, and confirming this with enzyme assay in leukocytes. Genetic analysis is also a frequently referred analysis but high incidence of novel “VUS” changes sometimes makes the interpretation difficult (43, 44). Newborn screening was carried out at in Illinois using multiplexed tandem mass spectrometry for five different LSDs including Niemann-Pick A/B disease. Of the 219.973 infants screened, two were found to have Niemann-Pick A/B showing that NBS provides the potential for early diagnosis and treatment (45).

### 6.4. Other clinical and laboratory assessments

The clinical and laboratory assessments highly suggestive of ASMD include histological tests that are performed to assess sphingomyelin accumulation (presence of characteristic large, lipid-laden foam cells present in the liver, spleen, lymph nodes, airways, and bone marrow), liver function tests (elevated transaminases) as primary effect of decreased ASM activity where inflammation and fibrosis are secondary effects, the lipid profile (low HDL cholesterol with high levels of LDL cholesterol and triglycerides), pulmonary function and lung imaging, skeletal radiographs, bone density measurement and clinical neurological and ophthalmological examinations, including direct ophthalmoscopy to assess presence of cherry-red spots (1, 6, 7, 42).

#### 6.4.1. Liver tests: biopsy, elastography, serum transaminases

Sphingomyelin (SM) storage in the liver, mainly in the liver-specific macrophages and Kupffer cells, is directly related to the deficiency of ASM, while the storage pattern of SM seems to reflect disease severity with higher amounts of SM and accumulation in hepatocytes besides Kupffer cells in liver biopsies of patients with lower residual enzyme activity (21, 46).

Although liver transaminases are often elevated in ASMD, the assessment of fibrosis or cirrhosis in the liver biopsies is considered more important, given its association with one of the clinical endpoints and the SM storage pattern in both Kupffer cells and hepatocytes (21, 42).

Transient elastography (fibroscan) is widely used in monitoring the progression of fibrosis in patients with HCV and proved to be a solid alternative for invasive and risky liver biopsies (47) and considered useful in detecting fibrosis in ASMD patients (5, 42).

#### 6.4.2. Lung tests: biopsy, HRCT, radiography, spirometry

Lung biopsies indicate lipid laden cells along with predominantly foamy macrophages located in the alveolar spaces and walls and in broncho-alveolar lavage fluid (29, 42, 48, 49). The interstitial inflammation and fibrosis occur in varying degrees (29, 42, 48, 49). High-resolution computed tomography (HRCT), the most reliable technique to investigate early signs of ASMD-dependent ILD, reveals a ground glass pattern, thickened interlobular septa and intralobular lines (29, 30, 42, 48, 49). Spirometry findings in ASMD patients are consistent with restrictive pulmonary disease (normal or decreased lung volumes and a decreased diffusion capacity) (27). However, while both HRCT and x-ray imaging indicate ILD-based abnormalities indicating, findings often do not correlate with impairment of lung function or clinical symptoms (13, 26, 42, 48) alongside the lack of correlation between pulmonary involvement on HRCT and spirometry-based pulmonary function (26).

#### 6.4.3. Spleen volume on imaging and platelet count

Spleen volume as measured by MRI is a possible biomarker in ASMD (13), as correlated positively with liver volume and triglyceride levels, and negatively with HDL levels, hemoglobin levels, white blood cell count and height in the presence of other disease specific signs and symptoms (13). In ASMD, low platelet counts due to splenomegaly is common and worsens over time as correlated with risk of bleeding (10, 17, 18, 42).

#### 6.4.4. Serum lipid profile

In ASMD patients, lipid profile is disruptive with high serum total cholesterol and LDL levels and low HDL levels (3), whereas the clinical relevance of the low HDL serum levels in relation to cardiovascular events remains unclear (42).

#### 6.4.5. Skeletal involvement

In ASMD, decreased bone marrow fat fractions on quantitative chemical shift imaging (QCSI) as well as decrease in bone mineral content (BMC), and bone mineral density (BMD) on dual energy x-ray absorptiometry (DEXA) scans have been described, possibly due to bone marrow infiltration by foamy macrophages (17, 24).

### 6.5. Potential biomarkers

The potential biomarkers regarding the burden of the disease useful in diagnosis as well as in monitoring the treatment responses include sphingomyelin and its derivatives, macrophage markers, exercise tolerance markers and QoL (6, 7, 37, 42).

#### 6.5.1. Sphingomyelin and its derivatives

In ASMD, due to impaired degradation of SM to ceramide, accumulated SM is converted into lysosphingomyelin (Lyso SM) (50). Lyso SM, as well as an analog of Lyso SM called Lyso SM 509/PPCS are considered possible biomarkers for ASMD since strongly elevated plasma levels were established in ASMD, as correlated with disease burden (37, 42, 50, 51). Simultaneous measurement of Lyso SLs (Lyso SM and Lyso SM 509 or Lyso SM 509/Lyso SM ratio) is considered to permit the distinction between the two diseases since the increase of Lyso SM and Lyso SM 509 in ASMD whereas of Lyso SM 509/Lyso SM ratio in NPD type C were noted to be higher due to a mild/no increase in Lyso SM in NPD type C (37, 51, 52). Nonetheless, Lyso-SLs are considered to be related to disease severity and to be more reliable biomarkers than oxysterols (37, 51).

#### 6.5.2. Macrophage markers

Several macrophage markers, through the intracellular lipid accumulation mediated activation of macrophages, are elevated in plasma of ASMD patients including chitotriosidase, chemokine CCL18, but with overlap with other LSDs (37, 42, 53). The chronic neurovisceral form has been suggested to be associated with higher biomarker levels than chronic visceral form, as correlated with the degree of visceral and pulmonary involvement and progressive neurological deterioration (37).

#### 6.5.3. Oxysterols

Oxysterols, oxygenated derivatives of cholesterol such as cholestane-3β, 5α, 6β-triol (C-triol) and 7-ketocholesterol (7-KC), are removed from the body during normal cholesterol metabolism, while they are increased in NPD C, chronic ASMD, and LALD (54, 55).

#### 6.5.4. Markers of exercise tolerance

The assessment of exercise tolerance is considered useful in ASMD patients as a functional marker of pulmonary, cardiac and musculoskeletal systems (42). The 6-minute walk test (6MWT) is the most commonly used exercise protocol in patients with ASMD (17). However, while it refers to a useful parameter enabling period assessment over time, the correlation between 6MWT and cardiopulmonary disease in ASMD is currently unknown (42).

#### 6.5.5. Markers of organ involvement

In relation to accompanying risk of cirrhosis, portal hypertension and variceal bleeding, hepatic disease is a considerable cause of morbidity and mortality in patients with chronic visceral disease (5, 7, 11, 14, 15). Splenomegaly can be massive (>20 multiples of normal spleen volume) and thus hypersplenism with increased risks for bleeding and splenic rupture is likely (7). In patients with chronic forms of ASMD infiltrative lung disease and atherogenic lipid profiles worsen with age (3, 18), while respiratory disease and organomegaly are independent contributors to mortality (27.7% for each) (14). The degree of splenomegaly correlates with short stature, atherogenic lipid profile and hematologic parameters in patients with chronic ASMD, and may be considered a surrogate marker for bleeding risk, infection risk, abnormal lipid profiles and liver fibrosis (15).

Progressive lung disease is a prevalent clinical feature of chronic ASMD as associated with decreased QoL and increased disease burden, and respiratory- related complications as well as mortality. This supports the use of DLCO and spleen volume as clinically relevant endpoints for disease burden in ASMD trials (15).

Accordingly, the lung diffusion capacity, spleen volume, platelet count and LDL-cholesterol, fibroscan-based liver fibrosis along with Lyso SM or Lyso SM 509/PPCS and 6MWT are considered the most promising biomarkers that correlate with pathophysiological process, treatment response and clinical events in ASMD (15, 42).

## 7. Disease monitoring and management

The current management of ASMD is based on alleviation of symptoms with treatments, interventions, and lifestyle modifications that address the QoL, morbidity and complications (7).

### 7.1. Monitoring assessments

Patients with chronic visceral and chronic neurovisceral ASMD require appropriate follow up and monitoring of multisystem manifestations for optimizing outcomes of ASMD by an interdisciplinary clinical team throughout childhood and adulthood (7).

The proposed monitoring strategy in ASMD patients are provided in Table 4 (7). The participating experts consider periodic assessment of the following parameters in chronic ASMD patients:

• Weight and linear growth and nutritional assessments in children (every 6–12 months)
• Hematologic abnormalities (yearly; coagulation profiles and CBCs for thrombocytopenia and bleeding risk with increasing age),
• Liver panels (every 6–12 months; transaminases, GGT, bilirubin, albumin, and prothrombin time/INR),
• Lipid profiles (yearly)
• Progression of liver fibrosis (ultrasound-based transient elastography, liver biopsy)
• Pulmonary function testing (yearly; DLCO, O_2_ saturation, and exercise capacity)
• Radiologic lung imaging (every 2–4 years; chest x-ray or low-radiation HRCT, due to discordance between symptoms/pulmonary function test findings and imaging findings),
• BMC and BMD in patients with signs of low bone density [every 2–4 years via dual energy x-ray absorptiometry (DEXA)],
• Age-appropriate neuropsychology assessments in pediatric and adolescent patients
• Peripheral neuropathy (more frequently during childhood in patients with 1 or 2 copies of the Q292K mutation, due to the association of this mutation with more severe neurologic abnormalities)

**Table 4:** Monitoring assessments, treatments/interventions/lifestyle modifications (7).

	Monitoring assessments	Treatments/interventions/life style modifications
Hematology	Yearly, coagulation profiles and CBCs for thrombocytopenia and bleeding risk with increasing age	Interventions (e.g., packing, cauterization) for frequent or excessive nose bleeds
Hepatic	Every 6–12 months; liver panel (transaminases, GGT, bilirubin, albumin, and prothrombin time/INR), progression of liver fibrosis (ultrasound-based transient elastography, liver biopsy)	Maintenance of nutrition and control of fluid retention Prohibition of alcohol use and hepatotoxic medications Non-selective beta blockers for prevention of esophageal varices bleeding Ammonia reduction for hepatic encephalopathy Antibiotics for spontaneous bacterial peritonitis Assessment of candidacy for liver transplant when needed
Spleen	Yearly, CBCs for thrombocytopenia and bleeding risk with increasing age	Caution regarding contact sports due to possibility of trauma-induced rupture
Cardiovascular	Lipid profiles (yearly)	Diet management and possibly statins (post-puberty) according to standard guidelines Stenting/CABG when necessary
Pulmonary	Pulmonary function testing (yearly; DLCO, O_2_ saturation, and exercise capacity) radiologic lung imaging (every 2–4 years; chest x-ray or low-radiation HRCT)	Supplemental oxygen and/or noninvasive positive pressure ventilation based on underlying abnormalities Bronchodilators for symptomatic pulmonary disease Aggressive management of all pulmonary infections Avoiding exposure to tobacco products
Skeletal	Weight and linear growth and nutritional assessments in children (every 6–12 months) BMC and BMD in patients with signs of low bone density (every 2–4 years via DEXA)	Exercise to prevent osteopenia Physical therapy as needed Standard dietary and lifestyle interventions to minimize bone loss Calorie intake adequate for growth
Neurological and cognitive	Age-appropriate neuropsychology assessments, peripheral neuropathy	Educational support Physical therapy

CBC, complete blood count; GGT, gamma-glutamyl transferase; INR, international normalized ratio; DLCO, diffusing capacity of the lung; HRCT, high-resolution computed tomography; DEXA, dual energy x-ray absorptiometry; CABG, coronary artery bypass graft.

### 7.2. Quality of life

In ASMD, QoL is mainly influenced by fatigue, dyspnea, and pain due to organ involvement (liver failure, severe splenomegaly, abdominal distension, compromised growth, compromised respiratory function with decreased DLCO, limited physical activity), while the disease is also suggested to negatively affect body image, self-esteem, and relationships with peers (13, 15, 17, 42, 56, 57). Accordingly, while not meet the definition of a standard biomarker, QoL questionnaires such as the Short Form 36 Health Survey (also known as SF36) or Health Assessment Questionnaire (HAQ) are considered useful tool in periodic assessment of QoL for disease monitoring (42).

### 7.3. Treatments, interventions and lifestyle modifications

Currently, the mainstay of therapy in ASMD is supportive symptom targeted approach to reduce morbidity and disease complications, and improve patient QoL (7).

The most common causes of disease-related morbidity and mortality are respiratory and liver failure in ASMD (1), while more pronounced visceral disease and the neurodegenerative phenotype are considered to contribute to earlier death in these patients (5, 11, 14).

Accordingly, the present expert panel considers pulmonary disease (infiltrative lung disease, respiratory infections, or insufficiency), liver disease (cirrhosis) and hematologic disease (thrombocytopenia, hemorrhage, splenic rupture, injury, postoperative hemorrhage, splenic vein tear, and gastrointestinal bleeding/varices bleeding) as the main causes of premature death in chronic ASMD (11, 13, 14, 17, 22). Hence, in patients with chronic visceral and chronic neurovisceral ASMD the treatment goals should include reducing splenomegaly and improving liver function and respiratory status, with the ultimate goal of decreasing serious morbidity and mortality (14). The proposed treatments, interventions and lifestyle modifications based on organ-specific involvement are provided in Table 4.

Treatment options for liver disease are limited including symptom management in patients with end-stage liver disease (control of edema and ascites, prevention of esophageal varices bleeding, hepatic encephalopathy and spontaneous bacterial peritonitis, vaccinations and nutritional maintenance), while liver transplantation is performed in several ASMD patients depending on the degree of complications resulting from cirrhosis (7).

No satisfactory treatment options are available for splenomegaly and splenectomy is generally contraindicated due to risk of liver disease exacerbation and progressive respiratory insufficiency. While in cases with massive splenomegaly, compression symptoms, and severe unsustainable hypersplenism, particularly in case of planned bone marrow transplantation, or in cases of emergency surgery for splenic trauma, necrosis, or rupture then partial splenectomy is a potential option (7).

While transfusions may be needed in extreme cases of bleeding, interventions such as nasal packing and cauterization are more commonly applied for patients with frequent or severe nose bleeds (7). Although reduction in liver and spleen size has been reported after bone marrow transplantation, complications secondary to the transplant procedure may be severe (1, 58).

Treatment options for pulmonary involvement are limited with use of oxygen therapy and bronchodilator for symptomatic pulmonary disease, prevention of first-and second-hand exposure to tobacco smoke, appropriate management of pulmonary infections along with administration of preventative vaccinations for pediatric and adult patients (7).

In neurological forms, educational support and physical therapy are performed on demand (7). Family counseling and the early involvement of palliative care teams are recommended for individualization of therapy and improved QoL in infantile neurovisceral ASMD patients. Nutritional support, physiotherapy, and spasticity management are amongst the other interventions (7).

Treatment and lifestyle changes include dietary modifications for adolescents and adults, lipid-lowering therapy for dyslipidemia, interventions (i.e., weight bearing exercise) to prevent/delay bone loss, physical therapy for joint and limb pain, as well as no restriction of dietary fat and cholesterol to ensure adequate caloric intake and optimal growth in children with severe growth restriction (7).

Supportive services (patient, and disease support groups, social services, family counseling) may offer improved quality of care and QoL (7). Genetic counseling is important to inform patients and families on the autosomal recessive inheritance pattern, carrier status, and potential impact on future offspring and siblings. When both SMPD1 pathogenic variants are identified in a family, carrier testing for at-risk relatives and prenatal diagnosis are possible. Prenatal diagnosis is also likely via testing for ASM enzyme activity (7).

Nonetheless, two registrational trials for olipudase alfa, the first etiology-specific treatment for ASMD in children and adults (7, 12, 15), revealed that 52-week olipudase alfa treatment was well-tolerated along with significantly improved disease pathology across a range of clinically relevant endpoints [pharmacokinetics, spleen and liver volumes, lung diffusing capacity (DLCO), lipid profiles, and height] in children with chronic ASMD (59) as well as associated with significant improvement in clinically relevant endpoints (DLCO, spleen and liver volumes, liver function/sphingomyelin content, pulmonary imaging/function, platelet levels, lipid profiles, and pharmacodynamics) compared with placebo in adults with chronic ASMD (60).

Basically, the metabolic disease specialists provide the medical care for patients with ASMD, while primary care providers and other specialists (e.g., pediatricians, hematologists, gastroenterologists, cardiologists, pulmonologists) can also be part of a team approach to patient care (7). The participating experts emphasize the consultation and communication between physicians involved in ASMD management to enable the familiarity with the routine care for the multisystem impact of the disease (7), while consider the rarity of pediatric metabolic disease specialists, availability of only symptomatic treatment, progression of liver and pulmonary disease, complication related to thrombocytopenia, presence of limited available evidence on progression, clinically significant events and mortality are the main challenges in the management of ASMD. While ERT is likely to become available for patients with ASMD soon, it is also unlikely to reverse the neurological impairment, emphasizing the need for future research that addresses widespread enzyme delivery to the brain, and novel alternatives to enzyme infusions such as gene therapy and small molecule approaches (1, 7).

## 8. Conclusions

ASMD is a rare multi-system disease with a wide spectrum of clinical manifestations in accordance with the continuum of disease severity driven by the neurological involvement, the extent of systemic disease, and disease progression as well as the heterogeneity of SMPD1 mutations. The participating pediatric metabolism experts consider the clinical suspicion of ASMD by the physician to be of utmost importance in prevention of diagnostic and therapeutic delay in these patients and strongly suggest the use of a diagnostic algorithm combined with DBS assay and genetic mutation analysis in timely diagnosis of ASMD in patients presenting with hepatosplenomegaly.

The experts emphasize the need for appropriate follow up and monitoring of multisystem manifestations for optimizing outcomes of ASMD by an interdisciplinary clinical team with appropriate consultation and communication between physicians. In anticipation of the introduction of ERT, raising awareness of the disease among physicians to prevent diagnostic delay and further investigation addressing natural history of ASMD across the disease spectrum, potential presenting characteristics with high index of suspicion, as well as biomarkers and genotype-phenotype correlations suggestive of poor prognosis seem also important in terms of implementation of best practice patterns. Accordingly, this expert opinion review, addressing the clinical spectrum and natural course of disease, clinical manifestations and diagnostic odyssey and disease monitoring with appropriate algorithms, provides a practical guidance document that would assist clinicians for best clinical practice in the management of ASMD.
